# Unreasonable reasons: normative judgements in the assessment of mental capacity

**DOI:** 10.1111/j.1365-2753.2012.01914.x

**Published:** 2012-09-21

**Authors:** Natalie F Banner

**Affiliations:** Centre for the Humanities and Health, King's College LondonLondon, UK

**Keywords:** clinical judgement, decision making, mental capacity, philosophy of medicine, psychiatry, rationality

## Abstract

The recent Mental Capacity Act (2005) sets out a test for assessing a person's capacity to make treatment choices. In some cases, particularly in psychiatry, it is unclear how the criteria ought to be interpreted and applied by clinicians. In this paper, I argue that this uncertainty arises because the concept of capacity employed in the Act, and the diagnostic tools developed to assist its assessment, overlook the inherent normativity of judgements made about whether a person is using or weighing information in the decision-making process. Patients may fail on this criterion to the extent that they do not appear to be handling the information given in an appropriate way, on account of a mental impairment disrupting the way the decision process ought to proceed. Using case law and clinical examples, I describe some of the normative dimensions along which judgements of incapacity can be made, namely epistemic, evaluative and affective dimensions. Such judgements are complex and the normative standards by which a clinician may determine capacity cannot be reduced to a set of criteria. Rather, in recognizing this normativity, clinicians may better understand how clinical judgements are structured and what kinds of assumption may inform their assessment.

## Introduction

Determining whether or not a patient is mentally capable of making a decision about his or her treatment or welfare is one of the most conceptually and ethically challenging areas of clinical practice, particularly in psychiatry and geriatrics. The Mental Capacity Act (MCA) (2005) 1 in England and Wales sets out criteria for a test of capacity,^1^ aiming to provide guidance for clinicians on striking a balance between preserving the autonomy of the patient while allowing care to be provided in the ‘best interests’ of those who lack the capacity to make their own decisions 2. The implementation of the MCA has heralded a surge of interest in the concept of mental capacity, its role in medical decision making and its assessment in health care 3.

In practice, the balance between autonomy and protection can be difficult to achieve, and there are cases where it is not clear whether a person possesses or lacks capacity with respect to a particular decision. Here, the assessment may depend on how clinicians interpret the test for capacity: patients may appear technically to fulfil the criteria while clinical intuition pulls in the other direction. Using a conceptual analysis of capacity and its methods of assessment, I argue that resulting uncertainty in clinical judgement arises because the descriptive criteria for capacity overlook the intrinsic normativity of the judgement. Case law and clinical examples provide the background to my exploration of what kinds of normative judgements are involved in capacity assessment.

## The MCA

The test of capacity in the MCA reflects capacity legislation in many other countries such as the United States, Canada and Australia. Details of the tests and procedures vary slightly between jurisdictions but all employ a similar notion of capacity as a time- and decision-specific ability to make a choice regarding one's treatment, welfare or finances. The MCA was developed with the intention of avoiding undue medical paternalism by allowing individual patients to make their own choices if they are capable, irrespective of whether or not these choices seem wise to others or in the patient's own best interests 4. To this end, much emphasis has been placed on evaluating the *processes* a patient uses to make a decision rather than the *content* of the decision itself: ‘*What matters is* [the] *ability to carry out the processes involved in making the decision – and not the outcome*’ 5. This functional test of capacity reflects a common approach in health contexts regarding a person's capacity to consent to medical treatment, turning on the quality of understanding a patient demonstrates 6.

The MCA test of capacity stipulates that for the powers of the Act to apply, a patient must be suffering from a mental impairment that causes him to be unable to:

- understand information relevant to the decision;
- retain that information;
- use or weigh that information as part of the process of making the decision; or
- communicate his decision. 7

If an individual cannot demonstrate any one of these abilities in spite of efforts being made to assist the decision-making process, then he lacks capacity. The importance of the first stage of the test – that an individual is suffering from an impairment or disturbance to the mind or brain – cannot be overstated. If there is no suspicion of a mental impairment, the individual is free to decide as he or she likes: ‘*the patient's right of choice exists whether the reasons for making that choice are rational, irrational, unknown or even non-existent*’ 8. If, on the other hand, a mental impairment is suspected then the second stage of the test, which scrutinizes the decision-making process in some depth, can be applied. The MCA is clear in its intention to avoid discriminating against patients merely on the basis of a diagnosis of mental disorder or learning disability, but psychiatric patients are nonetheless at a greater risk of being deemed to lack capacity than other clinical populations, in virtue of their diagnosis 9,10: ‘*until recently it was commonly presumed that serious mental illness, by definition, rendered a patient incapable of consenting to treatment*’ 11.

Surrogate decision making is occasionally justified irrespective of capacity, if there is a perceived risk of harm severe enough to warrant detention under a section of the Mental Health Act (MHA) 2007. The interface between the two acts is not entirely clear, and because the MCA is relatively new, few precedents common law have yet been established determining how the different pieces of legislation should interact 12,13. However, most cases do not fall within the jurisdiction of the MHA and for these, ascertaining whether a patient passes the capacity test can be extremely challenging. Consider the following examples, drawn from a discussion of the notion of ‘competence’, which I shall take here to be equivalent to capacity 14:

1. A severely anorexic patient refuses nasogastric feeding. He understands what the treatment involves, and that it could save his life. He appreciates that it is his decision to make and despite agreeing that without the procedure he risks death, he will not consent.
2. A patient with chronic depression needs surgery to remove an ovarian tumour. Doctors tell her that without surgery she is likely to die. She is able to grasp this information and discuss the options, but attaches no emotional significance to the decision 15, is ambivalent about whether she lives or dies and thus will not consent to the operation.

How ought clinicians to approach these cases? Both patients are diagnosed with a mental disorder that affects their thinking, but does this undermine capacity? Reflecting on the capacity test criteria, in both instances, the patients evidently understand the treatment information and appear to use and weigh it *insofar* as they are capable of discussing the options and the likely risks and outcomes involved, as well as communicating a decision not to consent. However, the authors of the commentary argue that, as clinicians, they would be reluctant to allow that either patient possessed the capacity to make the treatment choice. Despite apparently ticking the boxes for the capacity criteria, there is a strong clinical intuition that something about these patients’ decision making is not right, and furthermore that it is not right *because* of their illnesses. I suggest that we need a better grasp of what capacity is and how it has been conceptualized to identify the source of this tension between clinical intuition and the criteria for the test of capacity. It is therefore instructive to examine what frameworks for understanding capacity are available to clinicians and explore how it is assessed in practice.

## Empirical approaches to capacity

Capacity is a legal, clinical, ethical and social construct 16, yet it has primarily been conceptually explored in empirical studies. Charland distinguishes two aspects of capacity, arguing that it consists of both a descriptive and a normative form 17. The descriptive dimension concerns what capacity is and how it can be measured, understanding of which aims to provide objective definitions of what it is to possess or lack capacity, and to develop diagnostic tools to help clinicians make this determination. The second dimension is an ethically normative one, concerning whether or not a person ought to retain his right to personal autonomy: this aspect is determined by the legal and ethical framework within which the assessment is undertaken. Normativity here is construed in terms of the moral obligations or imperatives incumbent upon a person making a decision, and this ethically oriented dimension of capacity only comes into play if he or she is making extremely harmful choices that put the self or others at risk.

Carving up the dimensions of capacity assessment in this way provides an insight into the way empirical research has proceeded. If we are seeking to *assess* mental capacity, the descriptive component appears to fulfil this role sufficiently, and independently of the ethically normative dimension. Separating out the ethically normative issues from the descriptive project ensures that research into what capacity is and how it can be assessed can continue independently of the ethical complexities of balancing autonomy with protection.

The descriptive dimension of capacity has been largely conceptualized in empirical research through the ‘cognitive’ approach 18. This approach assumes that capacity can be operationalized through measuring its constituent psychological processes, which are taken to be, in principle, amenable to observation and measurement via the construction of behavioural indices. For example, the widely used MacArthur Competence Assessment Tool-Treatment (MacCAT-T) 19–21 uses a semi-structured interview format centred around the assessment of cognitive criteria. The MacCAT-T has been used to determine the prevalence of incapacity in general medical 22 and psychiatric settings 23 and it provides a high degree of inter-rater reliability 24. This highlights one of the obvious advantages of this approach: reliable criteria and testing procedures create a transparent process, reducing the risk of abuse or misplaced paternalism, and standardizing assessments across the board.

Such a methodology ostensibly provides certain benefits: ‘*the fact that these abilities are characterised as cognitive is usually deemed essential to the objectivity of the proposed operationalized standards for assessing them*’. The shift towards operationalizing criteria of psychological functioning, particularly for the diagnosis of mental illness, has been mirrored by progressive editions of the DSM-III onwards. Members of the DSM taskforce have explicitly argued for reducing the role of clinical judgement to ‘*remove items that cannot be determined reliably through patient self-reporting or through objectively observable signs of behaviours*’ 25. Reliance on the clinician's judgement is considered to be inferior compared with objective physiological markers and psychological assessments based on rating scales. To this end, the clinician is merely a trained impartial observer making descriptive judgements about a patient's mental states and cognitive functioning.

Despite acknowledging the difficulty in establishing a ‘gold standard’ for capacity assessment instruments, the cognitive conception relies on the assumption that capacity is an objectively measurable phenomenon 26. However, diagnostic tools are calibrated during their development by the judgement of an expert clinician, not a perspective-neutral measure of psychological functioning 27,28. When using such tools to assist in assessment, clinicians often reach different conclusions about capacity than when they are not guided by the cognitive concept of capacity 29,30. The cognitive bias in research has been criticized for underplaying the complexity of decision making and reducing assessment to measuring the operationalized elements of capacity 31,32. Such criticisms undermine not merely the level of refinement of the tools but also the assumption that capacity can be determined by ascertaining indices of patients’ mental functioning.

## Capacity assessments as normative judgements

The major conceptual flaw in cognitive accounts of capacity is that underpinning the assessment of the descriptive criteria for capacity is an intrinsically normative judgement. Specifically, assessing the criterion of using, weighing or balancing information involves the clinician making a judgement that hinges upon whether the patient is appropriating and using the information given in the way that he, in a sense I will seek to clarify, ought to. Importantly, this sense of how the patient ‘ought to’ use information is open to interpretation by clinicians, and it is my contention that this is what gives rise to disagreement in difficult cases.

The notion of ‘using or weighing information’ has not been much refined by case law or diagnostic tools. Assessors need to determine if a decision has been reached through a process that indicates the patient has taken account of the relevant information and options, and weighed this information in the balance. However, the clinician is operating from an epistemically limited position, and many of the factors that influence a patient's decision making are likely to be opaque from a third person perspective.

I suggest that determining if a patient is using or weighing the information he has been given is a matter of whether this information is having the *right* kind of impact on the decision process. That is to say, the information should occupy a normatively significant role in the patient's decision making. To use a simplified example, say I were to tell a person standing at a set of traffic lights that a lorry was hurtling along the road ignoring red lights, and I have every reason to believe he understands my utterances. He then proceeds to step directly into the lorry's oncoming path. Without being able to delve into his reasoning, I would judge that this person had failed to use or weigh the information I had given him, because he did not respond to that information in the way that he *ought* to (taken with the assumption that he had an interest in preserving his own life). There are of course numerous qualifications that could vindicate his move as reasonable or appropriate, but the point I am pressing is that making a judgement about whether a person is using or weighing information hinges on whether that information is perceived by an observer as being used in the right kind of way. The person's failure to use or weigh information is, in this case, indicated by the fact that his action was the wrong thing to do in those circumstances, as it would likely lead to his death or serious injury. When we invoke normative statements about what one ought to do or think, they make claims on us: they oblige, justify, constrain or guide intentions and actions and do not merely describe what we do 33. In other words, saying that someone is failing to use or weigh information is not a matter of describing impairments in his psychological functioning (although these may well impact on decision making), but is rather a judgement that there has been a normative failure to respond in an appropriate way to that information.

This sense of normativity has to be carefully cashed out if we are to avoid being too prescriptive about what kinds of responses constitute an appropriate use of information. Clearly, we must avoid a strongly prescriptive ‘ought’ that would imply a person is only using or weighing information if he makes a decision seen as ‘good’ from the clinician's perspective. I suggest the normative ‘ought’ here is considerably weaker: it does not prescribe the correct range of outcomes but rather circumscribes the kinds of decision processes that could reasonably follow from the information given. In this respect, there ‘ought’ to be appropriate or reasonable relations between the information a patient receives, what he values, believes and decides. If such relations are not in evidence or they seem unwarranted or inappropriate, then the person's decision making warrants further scrutiny and he may lack capacity.

This indicates that using or weighing information requires more than a demonstration by the patient that he is aware of the risks and consequences of the choice he makes, which could be assessed through descriptive measures of understanding and appreciation. It also requires that this information makes a difference to the decision process, which can only be assessed insofar as the information plays a normative role in shaping the decision. The distinction between descriptive and ethically normative components of capacity is thus misleading: it assumes that the *only* normative considerations relevant to capacity are ethical norms that impact on assessment after the fact of making a descriptive judgement about a person's capacity. However, the descriptive assessment about whether or not a person is using or weighing information is underpinned by a non-ethical normative judgement about the appropriateness of that decision-making process. This is an important point. Capacity cannot be determined using operational measures alone if we acknowledge that assessments are dependent a normative judgement about the way a patient is using or weighing information.

## Norms of judgement in capacity

Normative standards and judgements are an ordinary part of clinical practice, and acknowledging their role in capacity assessment need not undermine their reliability or introduce clinical bias. Indeed, many medical assessments are based on establishing whether or not patients conform to certain norms of physical or psychological functioning. For example, there is a normative implication to having a blood pressure above the normal range, which is that, in ordinary circumstances, one ought to take steps to reduce it for the sake of one's health. Where normative standards are universally agreed upon, they are conceptually and pragmatically unproblematic. However, I have argued that assessments of mental capacity involve an inherently normative judgement about what constitutes an appropriate or reasonable use or weighing of information, and it is by no means clear what normative standards discipline such judgements, or whether they might diverge between clinicians and patients. Understanding what the normative elements of capacity assessment are and how they are structured is acknowledged, in the conceptual literature at least, to be an increasingly important area of philosophical, legal and psychiatric research. How then are we to handle the normativity of using or weighing information, without being overly prescriptive about what constitutes reasonable or appropriate decision making?

The MCA is clear in its intent to focus assessment on the process used in coming to a decision, and not the content of the decision. To this extent, it could be argued that what matters for capacity is the internal rationality of the process, namely that the outcome is broadly consistent with the patient's beliefs and values 34. That is to say, irrespective of any external judgement of the outcomes of decision making, including the intermediate reasons given for a decision, if a decision is rational on the patient's own terms it could be deemed to result from a decision-making process indicative of capacity.^2^ A decision could therefore be identified as lacking capacity insofar as it does not reasonably follow from the patient's own previously expressed beliefs and values, irrespective of what these actually are. This would avoid capacity assessment imposing any external belief or value system upon the patient's decision-making process. However, both the case law and the clinical examples cited previously imply that when a mental impairment is suspected, a coherent, consistent process alone is not sufficient for capacity. In a case brought before the Court of Appeal, a chronically depressed woman with a diagnosis of borderline personality disorder refused to eat, to the extent that her life was in imminent danger. Her clear and rigorous articulation of her reasons for refusing to eat actually assisted the judge in deciding that her decision was caused by her mental illness, and as a result allowed compulsory treatment by way of nasogastric feeding to be given under the powers of the MHA: ‘*It is … this very self-awareness and acute self-analysis which leads me to doubt whether, at the critical time, she could be said to have made a true choice in refusing to eat*’ 35. Similarly, individuals suffering from *anorexia nervosa* are often insightful, coherent and able to understand the necessity of forcing a dangerously underweight, malnourished person to ingest food 36–38 but as suggested, doubts may be raised about such individuals’ capacity despite the internal rationality of their reasoning.

It is important to reiterate that in order for decision making to be scrutinized, first a mental impairment must be identified, and the assessment then focuses on whether this impairment undermines capacity: assessment cannot and should not be used to identify a mental impairment, or to adjudicate a mental illness diagnosis. Otherwise, the process risks a circularity that would deny capacity to anyone with beliefs and values believed unusual or harmful by a clinician.

In fact, normative judgements about the quality and content of an individual's beliefs, values and emotions do play a role in the assessment process, and I suggest exploring these three normative dimensions may provide a useful framework in which to examine the basis of clinical judgement about using or weighing information. Considering beliefs first, the presence of a mental disorder or impairment may cause a person to hold beliefs that are manifestly and objectively untrue: in one case, a female patient was admitted for an emergency Caesarean section, but she refused treatment because she denied that she was pregnant 39. A misperception of reality provided strong evidence that the patient lacked capacity, as she was either incapable or unwilling to acknowledge an incontrovertible fact that endangered her life. Delusions are also strongly associated with assessments of incapacity 40. In a judgement granting a hospital the right to override a patient's refusal for a medically necessary hysterectomy, the court stated: ‘*a compulsive disorder or phobia may prevent the patient's decision from being a true one, particularly if conditioned by some obsessional belief or feeling which so distorts the judgment as to render the decision invalid*’ 41. Here, the patient believed that she was childless and refused the treatment on the grounds that she wanted children. The fact that she had two grown-up children indicated her capacity to make that decision was impaired.

In these cases, we can understand the patients’ lack of capacity in terms of a failure to believe facts that they ought to, or holding beliefs about these facts that they ought not to hold, which impinge upon the decision process. They are committing an epistemic error that undermines their capacity to decide. It is tempting to think we could derive an epistemic standard by which to judge capacity from these kinds of cases, for example, that to be deemed to have capacity one ought to believe true facts about one's diagnosis and proposed treatment. However, I suggest that such an attempt would be misguided. First, and foremost, it would potentially threaten the commitment to pluralism in and freedom of beliefs, taken to be a central liberal ideal of our legislature, as it is not clear whether a demarcation can be made between incontrovertible ‘true’ facts one ought to accept, and those the failure of which to believe would not undermine one's capacity. Medical advice is given on the best available evidence of the time, and particularly with regard to treatment options, belief in their potential efficacy, risks and side effects may vary considerably. Demanding a clear epistemic standard would also lead to the question of whether having capacity requires that a patient ought to believe all the information given to him about his condition and the available treatment options. Psychiatric diagnoses are controversial and rejection of a medical opinion does not imply the patient is necessarily lacking insight or failing to acknowledge incontrovertible facts. The case of *Re C* reminds us that having a delusion need not undermine capacity: a patient diagnosed with schizophrenia believed he was a world famous doctor; he was suffering from gangrene but refused to consent to a medically advised amputation. The court decided that his delusion did not affect his decision-making capacity and he was competent to refuse 42. Yet, ‘*patients who refuse treatment in a psychiatric setting are particularly likely to be judged as lacking capacity*’ 16, indicating that in common practice at least, clinicians may consider treatment refusal to indicate a lack of capacity because patients are failing to acknowledge facts about their condition and the need for treatment: they are failing to believe things they ought to.

A patient's values may also play a normatively significant role in decision making, although making judgements about the legitimacy or otherwise of a patient's values is more controversial. Research into decision making in patients with *anorexia nervosa* reveals a complex interplay between positive evaluations associated with the anorexic identity, self-control and thinness, distorted beliefs about body shape and extreme fears of weight gain, to name but a few factors 36. While many psychological and biological factors contribute to the continued refusal to eat, specific evaluative commitments underpin patients’ reasoning and motivations: ‘*treatment refusal may occur, not because the patient wishes to die, but because of the relative unimportance of death and disability as compared to anorexia nervosa*’. These patterns of evaluation serve as highly significant weights in the decision-making process, providing justifications for making a choice to refuse treatment 43. Again, we might construe such extreme evaluations as a normative mistake, as patients are failing to respond appropriately to information that is crucially important if they wish to continue living (such as: without treatment you are likely to die), on account of the overriding values that are intrinsic to the eating disorder diagnosis. This is not to say that patients ought to hold particular values, or that it is never justified to place a relatively low value on one's own life: it is not clear that there is an evaluative standard that could differentiate capacity-undermining pathological influences from legitimate but unusual ones such as commitment to religious doctrine, for example 44. Nonetheless, it is important to identify that intrinsic to assessing whether a patient is using or weighing information is a normative judgement about the role his value system plays in his decision making.

Decisions made about treatment impact upon the person's life, health and relationships, and thus contain an important affective element 45. Decision-making ability can be impaired subtly by disorders in which a person is perfectly capable of fulfilling the capacity criteria as an abstract exercise in intellectual functioning, while attaching no affective significance to the process or outcome 46, as in the case of the chronically depressed patient discussed in example 2 above. At the other extreme, emotions may disproportionately influence the decision-making process and undermine capacity by overwhelming the decision process; anxieties and phobias can obviate important information if they ‘*paralyse the will and thus destroy the capacity to make a decision*’ 47. There is thus a further normative dimension to assessing decision making: the appropriateness or proportionality of affective response a person has to the information given.

The process of decision making can, in principle, be influenced by unusual or eccentric beliefs, values and emotions, without detriment to the presumption of capacity. Capacity may, however, be undermined when beliefs, values or emotions resulting from a mental impairment cause the individual to be unable to use relevant information about a proposed treatment in an appropriate way as part of their decision making. The immediate difficulty identified here is in characterizing what constitutes a normative breach, without being prescriptive about the way a patient ought or ought not to use the information given. I suggest that rather than attempting to provide robust criteria for making this assessment, acknowledging that the assessment is underpinned by more of a socially normative than a medical judgement is in itself a useful enhancement for clinical judgement. I have outlined three normative dimensions along which a person's decision-making process can be assessed, namely epistemic (concerning beliefs), evaluative (values) and affective (emotions). Although this is not an exhaustive list, it is intended to encourage clinicians to consider how their assessments of a patient's ability to use or weigh information are structured by normative judgements about the way information is handled. It is open to interpretation quite what the normative standards disciplining clinical judgements may look like, or what might constitute a failure to respond appropriately to information, but understanding where differences arise might assist clinicians in navigating this complex judgement. For example, the weight placed on medical expertise, diagnosis or risk statistics may be authoritative for the clinician but culturally variable for patients; the value placed on individual life may be superordinate or relative to the needs of the family or community; and the influence of anxiety on patients may be underestimated by clinicians for whom the treatments being offered are simple and routine, to name but a few.

## Conclusion

Clinical judgement is an inextricable part of the assessment process, despite the drive towards operationalizing criteria of cognitive functioning. Capacity assessment is based in part on the clinician's understanding of what counts as using or weighing information in the decision-making process. The cognitive conception upon which the test of capacity in the MCA is based overlooks the fact that determining this criterion requires a complex normative judgement to be made, regarding what decision processes could reasonably follow from the information provided, and whether a person's beliefs, values and emotions affect how this information is handled in reasonable or appropriate ways. Rather than attempting to minimize or ignore this inherent normativity, we should seek to provide an account of what standards discipline clinical judgements in such assessments, and how they might potentially differ. In this paper, I have begun to set out some of the normative dimensions along which judgements of capacity could be made. These are indefeasible and irreducible to a simple set of criteria, and I suggest that clinical judgement is enhanced by recognizing that it involves navigating a complex encounter in which clinicians play an active role, not as impartial observers of cognitive functioning but as participants in judgement guided by normative assumptions about what it means to engage successfully in a decision-making process. Acknowledging and attempting to understand better what guides these norms of judgement, and how they influence capacity assessments, could equip clinicians with a more sophisticated approach to assessing capacity than a descriptive view alone would permit.

## Footnotes

1 The term ‘capacity’ refers to a time- and decision-specific ability, whereas competence is a legal category that refers to the status of a person with regard to all decision making. However, in North American literatures, ‘competence’ is used synonymously with capacity.

2 A wealth of sociological literature addresses the asymmetry between a person's ‘subjective’ rationality and social norms or expectations, which may impact on how patients’ decisions in a medical context are perceived and understood by clinicians 48. The argument presented here complements such views by suggesting that medical judgements that seek to be objective are in fact underpinned by normative judgements.

## References

[1] Department of Constitutional Affairs (2007). Mental Capacity Act 2005.

[2] Raymont V (2002). ‘Not in perfect mind’ – the complexity of clinical capacity assessment. Psychiatric Bulletin.

[3] Owen GS, Freyenhagen F, Richardson G, Hotopf M (2009). Mental capacity and decisional autonomy: an interdisciplinary challenge. Inquiry.

[4] MCA (2005).

[5] Department of Constitutional Affairs (2007). Mental Capacity Act 2005: Code of Practice.

[6] Gillick v West Norfolk and Wisbeth Area Health Authority (1985).

[7] MCA (2005).

[8] *Re T (Adult: Refusal of Treatment)* (1992).

[9] Wong JG, Clare ICH, Holland AJ, Watson PC, Gunn M (2000). The capacity of people with a ‘mental disability’ to make a health care decision. Psychological Medicine.

[10] Cairns R, Maddock C, Buchanan A, David AS, Hayward P, Richardson G, Szmukler G, Hotopf M (2005a). Prevalence and predictors of mental incapacity in psychiatric in-patients. British Journal of Psychiatry.

[11] Cairns R, Maddock C, Buchanan A, David AS, Hayward P, Richardson G, Szmukler G, Hotopf M (2005b). Reliability of mental capacity assessments in psychiatric in-patients. British Journal of Psychiatry.

[12] Richardson G (2010). Mental capacity at the margin: the interface between two Acts. Medical Law Review.

[13] Owen G, Szmukler G, Richardson G, David AS, Hayward P, Rucker J, Harding D, Hotopf M (2009). Mental capacity and psychiatric inpatients: implications for the new mental health law in England and Wales. British Journal of Psychiatry.

[14] Culver CM, Gert B, Radden J (2004). Competence. The Philosophy of Psychiatry: A Companion.

[15] Rudnick A (2002). Depression and competence to refuse psychiatric treatment. Journal of Medical Ethics.

[16] Hotopf M (2005). The assessment of mental capacity. Clinical Medicine.

[17] Charland LC (2001). Mental competence and value: the problem of normativity in the assessment of decision-making capacity. Psychiatry, Psychology and Law.

[18] Appelbaum PS, Roth LH (1982). Competency to consent to research. A psychiatric overview. Archives of General Psychiatry.

[19] Appelbaum PS, Grisso T (1995). The MacArthur Treatment Competence Study. I: mental illness and competence to consent to treatment. Law & Human Behavior.

[20] Grisso T, Appelbaum PS, Mulvey EP, Fletcher KL (1995). The MacArthur treatment competence study. II: measures of abilities related to competence to consent to treatment. Law & Human Behavior.

[21] Grisso T, Appelbaum PS, Hill-Fotouhi C (1997). The MacCAT-T: a clinical tool to assess patients’ capacities to make treatment decisions. Psychiatric Services.

[22] Raymont V, Bingley W, Buchanan A, David AS, Hayward P, Wessely S, Hotopf M (2004). Prevalence of mental incapacity in medical inpatients and associated risk factors: cross-sectional study. The Lancet.

[23] Vollman J, Bauer A, Danker-Hopfe H, Helmchen H (2003). Competence of mentally ill patients: a comparative empirical study. Psychological Medicine.

[24] Okai D, Owen G, McGuire H, Singh S, Churchill R, Hotopf M (2007). Mental capacity in psychiatric patients: systematic review. British Journal of Psychiatry.

[25] Kupfer DJ, First MB, Reggier DA (2002). A Research Agenda for DSM-V.

[26] Kim SYH (2006). When does decisional impairment become decisional incompetence? Ethical and methodological issues in capacity research in schizophrenia. Schizophrenia Bulletin.

[27] Janofsky JS, McCarthy RJ, Folstein MF (1992). The Hopkins Competency Assessment Test: a brief method for evaluating patients’ capacity to give informed consent. Hospital & Community Psychiatry.

[28] Bean G, Nishisato S, Rector NA, Glancy G (1994). The psychometric properties of the competency interview schedule. Canadian Journal of Psychiatry.

[29] Vellinga A, Smith JH, van Leeuwen E, van Tilburg W, Jonker C (2004). Instruments to assess decision-making capacity: an overview. International Psychogeriatrics.

[30] Mukherjee S, Shah A (2001). The prevalence and correlates of capacity to consent to a geriatric psychiatry admission. Aging and Mental Health.

[31] Breden TM, Vollmann J (2004). The cognitive based approach of capacity assessment in psychiatry: a philosophical critique of the MacCAT-T. Health Care Analysis.

[32] Higgs R (2004). The contribution of narrative ethics to issues of capacity in psychiatry. Health Care Analysis.

[33] Korsgaard CM (1997). The Sources of Normativity.

[34] Kennedy I (1997). Commentary on Re MB (Medical Treatment). Medical Law Review.

[35] B v Croydon Health Authority (1995).

[36] Tan J, Hope T, Widdershoven G, Hope T, McMillan J (2008). Treatment refusal in anorexia: a challenge to current concepts of capacity. Empirical Ethics in Psychiatry.

[37] Tan J, Stewart A, Fitzpatrick R, Hope T (2006). Competence to make treatment decisions in anorexia nervosa: thinking processes and values. Philosophy, Psychiatry & Psychology.

[38] Tan J, Hope T, Stewart A (2003). Competence to refuse treatment in anorexia nervosa. International Journal of Law & Psychiatry.

[39] Norfolk & Norwich Healthcare (NHS) Trust v W (1996).

[40] Owen GS, David AS, Richardson G, Szmukler G, Hayward P, Hotopf M (2009b). Mental capacity, diagnosis and insight in psychiatric in-patients: a cross-sectional study. Psychological Medicine.

[41] Trust A and Trust B v H (An Adult Patient) (2006).

[42] Re C (Adult: Refusal of Medical Treatment) (1994).

[43] Martin AM (2007). Tales publicly allowed: competence, capacity and religious beliefs. Hastings Center Report.

[44] Holroyd J, Radoilska L (2012). Clarifying capacity: values and reasons. Autonomy and Mental Disorder.

[45] Charland LC (2008). Decision-making capacity. The Stanford Encyclopedia of Philosophy.

[46] Charland LC (2006). Anorexia and the MacCAT-T test for mental competence: validity, value, and emotion. Philosophy, Psychiatry & Psychology.

[47] Re MB (Medical Treatment) (1997).

[48] Buetow S (2007). Non attendance for health care: when rational beliefs collide. The Sociological Review.

